# Text-Book of Medicine

**Published:** 1902-03

**Authors:** 


**Text-Book of Medicine.** Edited by GEORGE ALEXANDER GIBSON, M.D. Two volumes. Vol. I. Pp. xvi., 824. Vol. II. Pp. xvi., 909. Edinburgh: Young J. Pentland. 1901.

The scheme and scope of this *Text-Book of Medicine* are excellent for the purposes of the student and general practitioner. The editor has gathered round him a number of well-known authorities in the several departments in which they contribute, each one possessing the width of scientific and practical knowledge of his subject that is essential to the writing of clear, concise, and practical articles. The advantages of a text-book produced by such collaboration are obvious; the drawback too often consists in the want of due proportion between its several parts, and herein Dr. G. A. Gibson has displayed his skill as an editor, for judging this work as a whole, he has succeeded in giving us a well-balanced text-book, at once readable, authoritative and complete.

We cannot refer even briefly to the part taken by each of the many contributors, but must content ourselves with noting a few as samples of the contents.

The first volume opens with a preliminary discussion of general etiological and pathological problems—an article from the pen of the late Prof. Kanthack, left by his unfortunate death to be completed by Prof. Sims Woodhead. Such problems as immunity, immunisation, toxæmia, are comprised in this introduction, and the latest views are succinctly stated. The infectious fevers occupy the next 250 pages, and in this section we were especially attracted by the beautifully illustrated and masterly contributions of Dr. Patrick Manson on "Malaria"

and on "Diseases caused by Animal Parasites"; Sir J. W. Moore on "Epidemic Influenza"; and that on "Gout" by A. P. Luff, and "Diabetes" by R. T. Williamson.

In volume ii., Prof. Ralph Stockman is excellent in his articles on "The Hæmapoietic System." Dr. G. A. Gibson's contributions on "Diseases of the Heart" leave nothing to be desired. Sir Thomas Lauder Brunton writes on "Nervous Diseases of the Heart," and Prof. J. Rose Bradford on "Diseases of the Kidneys."

The other portions of the work are dealt with by equally well-known writers; and though we cannot mention all, it must not be inferred that their work has seemed less acceptable or less instructive, for the whole work is admirable.

